# Association between disruption of CD4 receptor dimerization and increased human immunodeficiency virus type 1 entry

**DOI:** 10.1186/1742-4690-3-31

**Published:** 2006-06-08

**Authors:** Rachel Bourgeois, Johanne Mercier, Isabelle Paquette-Brooks, Éric A Cohen

**Affiliations:** 1 Department of Microbiology and Immunology, Université de Montréal, Montreal, Quebec, Canada; 2 Laboratory of Human Retrovirology, Institut de Recherches Cliniques de Montréal, Montreal, Quebec, Canada

## Abstract

**Background:**

Human immunodeficiency virus (HIV) enters target cells by a membrane fusion process that involves a series of sequential interactions between its envelope glycoproteins, the CD4 receptor and CXCR4/CCR5 coreceptors. CD4 molecules are expressed at the cell surface of lymphocytes and monocytes mainly as monomers, but basal levels of CD4 dimers are also present at the cell surface of these cells. Previous evidence indicates that the membrane distal and proximal extracellular domains of CD4, respectively D1 and D4, are involved in receptor dimerization.

**Results:**

Here, we have used A201 cell lines expressing two CD4 mutants, CD4-E91K, E92K (D1 mutant) and CD4-Q344E (D4 mutant), harboring dimerization defects to analyze the role of CD4 dimerization in HIV-1 entry. Using entry assays based on β-lactamase-Vpr or luciferase reporter activities, as well as virus encoding envelope glycoproteins derived from primary or laboratory-adapted strains, we obtained evidence suggesting an association between disruption of CD4 dimerization and increased viral entry efficiency.

**Conclusion:**

Taken together, our results suggest that monomeric forms of CD4 are preferentially used by HIV-1 to gain entry into target cells, thus implying that the dimer/monomer ratio at the cell surface of HIV-1 target cells may modulate the efficiency of HIV-1 entry.

## Background

HIV-1 is an enveloped virus that gains entry into target cells by mediating the fusion of viral and cellular membranes. The first step of this fusion process involves the binding of gp120, the surface subunit of the viral envelope glycoprotein (Env), to its primary receptor, CD4. This interaction induces conformational changes in both proteins that lead to subsequent binding of gp120 to its coreceptor, CCR5 or CXCR4. Coreceptor binding is thought to next trigger major structural rearrangements in the transmembrane glycoprotein gp41 subunit, including the formation of a triple-stranded coiled-coil that enables the hydrophobic fusion peptide at the N-terminus of gp41 to insert into the target cell membrane. The triple-stranded coiled-coil then bends back on itself, forming a six helix bundle that brings the viral and target cell membranes into close proximity and allows membrane fusion (reviewed in reference [1]).

CD4 is a transmembrane glycoprotein that is mainly expressed by T-lymphocytes, monocytes/macrophages and dendritic cells. The CD4 receptor normally functions as a coligand and coreceptor of the major histocompatibility complex class II (MHC II) molecule during T cell recognition of a foreign antigen where, in fact, it stabilizes the MHC II-peptide-T-cell receptor complex and initiates intracellular signal transduction leading to T cell activation [2]. CD4 is predominantly expressed as a 55 kDa monomer, but CD4 dimers and tetramers (110 and 220 kDa) have also been found to be expressed at the cell surface of T lymphocytes and monocytes/macrophages [3-5]. The equilibrium established between these different forms may vary according to the cell type, the activation state of the cell and the redox conditions at the plasma membrane [3,6,7]. For instance, binding of CD4 to its oligomeric ligands, MHC II and gp120, is believed to induce the formation of CD4 oligomers [8], which in turn, trigger the activation of the CD4 cytoplasmic tail-associated tyrosine kinase p56^lck^, by *trans*-phosphorylation [9]. The subsequent signaling cascade was found to induce the nuclear translocation of the AP-1 and NF-κB transcription factors, promoting T-cell activation [10].

The extracellular portion of CD4 is composed of four immunoglobulin (Ig)-like domains, designated D1 to D4; D4 being the membrane proximal domain. Crystallographic studies have provided structural evidence indicating that D4 is involved in dimerization of the receptor. More precisely, residues in the CC' loop and the CDR3-like region of the D4 domain were shown to be part of a putative dimer interface, with residues Q344 and Q344' having the potential to be linked by a hydrogen-bond [11]. In fact, it was shown that mutation of this highly conserved glutamine residue by a glutamic acid (Q344E) impaired the dimerization of the receptor [4]. In addition to the D4 region, two other domains have been proposed to contribute to CD4 dimerization. First, structural and functional evidence suggest that the D1 domain is implicated in the formation of CD4 dimers. The negatively charged CDR3-like region and the positively charged CC' loop of D1 are involved in electrostatic interactions between two CD4 molecules [12]. Moreover, Briant *et al*. have found that the E91K, E92K mutations in D1 reduce the capacity of the receptor to induce NF-κB nuclear translocation following HIV-1 binding, thus suggesting that these mutations could reduce CD4 dimerization levels [13]. Finally, disulfide bonds in D2 also appear to play an important role in the formation of CD4 dimers since treatment with reducing agents was shown to strongly attenuate receptor dimerization [6].

Although CD4 dimers have proven to be important for the biological functions of the receptor [4,13], little is known regarding the role of CD4 dimerization during HIV-1 entry. In this study, we provide evidence suggesting that disruption of CD4 dimerization increases HIV-1 entry efficiency.

## Results

### Characterization of CD4 dimerization mutants

**Figure 1:**
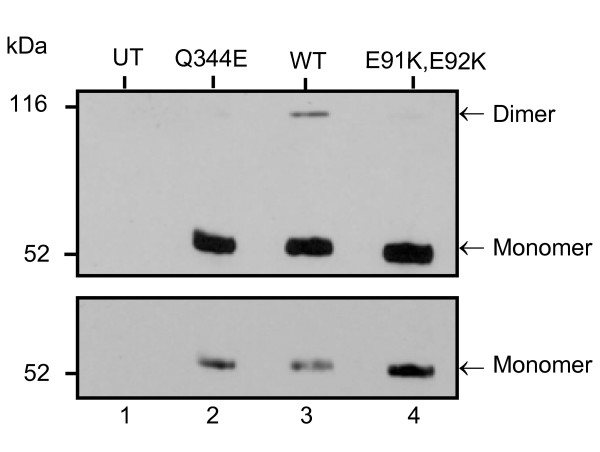
**Immunoblot analysis of CD4 dimerization levels of CD4-WT and mutants**. HEK 293 cells were transfected with SVCMV CD4-Q344E (lane 2), SVCMV CD4-WT (lane 3) and SVCMV CD4-E91K, E92K (lane 4) by a calcium phosphate method. Cells were lysed 48 hours post-transfection. Equivalent amounts of cell lysates were separated by SDS-PAGE, in a non-reducing Laemmli buffer and transferred onto a nitrocellulose membrane. Immunoblot analysis was performed using a CD4-specific polyclonal Ab. The lower panel represents a shorter exposition of the same membrane, showing unsaturated bands of CD4 monomers. This is a representative result of 5 independent experiments. (UT) represent the untransfected negative control.

To examine the role of CD4 dimers in HIV-1 entry, we made use of two CD4 mutants, namely CD4-E91K, E92K and CD4-Q344E that harbored mutations in two distinct regions of the molecule involved in CD4 dimerization. As a first step, we needed to confirm directly the putative dimerization defect of the CD4-E91K, E92K (D1) mutant since this defect was never directly demonstrated with this mutant. As a control, we used the CD4-Q344E (D4) mutant since our laboratory had previously reported that this mutant displayed a dimerization defect [4]. Figure 1 shows an anti-CD4 immunoblot of total cell lysates from human embryonic kidney (HEK) 293 cells, transiently expressing CD4-WT and CD4 dimerization mutants. Cells were lysed in a Nonidet-P40/Triton X-100 (NP-40/Triton) buffer containing 10 mM N-ethylmaleimide (NEM), a sulfhydryl blocking reagent, to eliminate dimerization artifacts due to disulfide bond formation after lysis. As expected, we detected a five-fold reduction of CD4 dimer levels for both mutants as compared to CD4-WT.

### Generation of A201 CD4 cell populations

**Figure 2:**
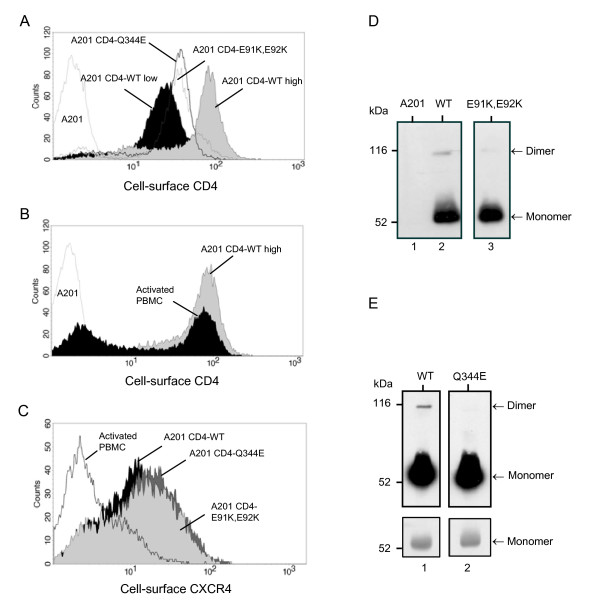
**Generation and characterization of A201 cell lines expressing CD4-WT and dimerization mutants**. (A) A201 CD4-WT, A201 CD4-E91K, E92K and A201 CD4-Q344E cell populations were stained with the anti-CD4 mAb OKT4 and a FITC-conjugated secondary Ab. CD4 expression levels at the cell surface were determined by flow cytometry. The A201 cell line was used as a negative control. (B) A comparison of CD4 cell surface expression in A201 CD4-WT high cell population and PHA-activated PBMC is shown. (C) Flow cytometric analysis of CXCR4 cell surface expression in the different selected A201 CD4 cell populations and PHA-activated PBMC was performed after staining the cell surface with the anti-CXCR4 mAb 12G5 and a FITC-conjugated secondary Ab. (D and E) Cell lysates from the different A201 CD4 cell populations were normalized for total CD4 amounts and then immunoprecipitated with the anti-CD4 mAb OKT4. Immunocomplexes were then resuspended in non-denaturing Laemmli buffer and separated by SDS-PAGE. Immunoblot analysis was then carried out as described in Figure 1. The A201 cell line was used as a negative control. Panels of the same section show separated lanes of the same gel. The lower panels in E represent a shorter exposure of the same membrane, showing unsaturated bands of CD4 monomers. These results are representative of 3 independent experiments.

To test the effect of these CD4 dimerization mutants on viral entry efficiency, we generated stable cell lines by transfecting A201, a human CD4 negative T cell line [14], with expression vectors encoding CD4-WT or the dimerization mutants. Populations of CD4-positive clones were selected by immunofluorescence cell sorting and then subjected to hygromycin selection to maintain CD4 expression. As cell surface expression of CD4 was fluctuating over time, we selected two control populations of A201 CD4-WT cells that had lower and higher CD4 levels than the A201 CD4-E91K, E92K and A201 CD4-Q344E cells (Figure 2a). Given that overexpression of CD4 could compensate for a viral entry defect, we compared cell surface CD4 expression in the A201 CD4 cell populations with that of PHA-activated PBMC. Flow cytometry analysis revealed that all selected cell populations expressed physiological levels of CD4 (Figure 2b and data not shown). Surface expression of the coreceptor CXCR4 was also measured, using the mAb 12G5. All selected A201 CD4 cell populations had comparable levels of surface CXCR4, and these levels were higher than those observed on activated PBMC (Figure 2c). Thus, CXCR4 expression is not a limiting factor for viral entry in the A201 cell transfectants.

The CD4 dimer to monomer ratio was then evaluated in the different A201 CD4 cell populations to confirm the dimerization defect of the D1 and D4 mutants in this cell type. Since levels of CD4 dimers were substantially lower in A201 CD4 transfectants than in 293 cells, larger amounts of lysate had to be analyzed in order to detect dimeric forms. Consequently, non specific background was increased, making direct analysis of CD4 dimerization by western blot difficult. To bypass this limitation, we immunoprecipitated similar amounts of CD4 or CD4 dimerization mutants prior to the immunoblot analysis. Here again, we could observe an important decrease in the dimerization capability of CD4-E91K, E92K and CD4-Q344E mutants as compared to CD4-WT (Figure 2d and 2e). Our results are consistent with those of others who have already shown that CD4 monomers are predominantly expressed in lymphocytes and monocytes [3].

### Disruption of CD4 dimerization is associated with increased viral entry efficiency

**Figure 3:**
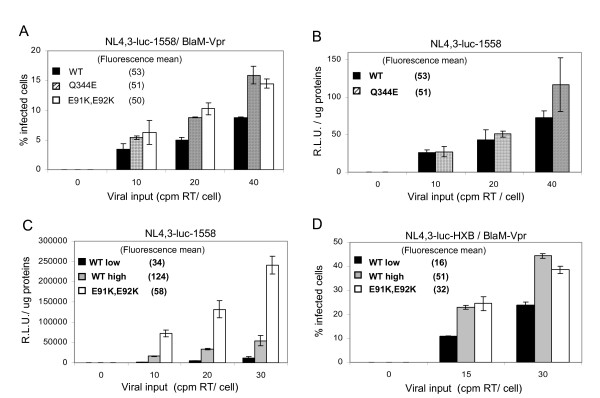
**Disruption of CD4 dimerization is associated with increased HIV-1 entry into target cells**. A201 CD4-WT, A201 CD4-E91K, E92K and A201 CD4-Q344E cell populations were incubated 3 hours with increasing viral inputs, as determined by standard reverse transcriptase (RT) assays, of NL4, 3-luc-1558 (A, B and C) and NL4, 3-luc-HXB (D) virions containing BlaM-Vpr fusion protein. (A and D) Following a 3 hours adsoption period, a portion of cells were treated with CCF2-AM and analyzed by flow cytometry, 16 hours later. Each assay was done in duplicate and results are representative of 3 and 2 experiments, respectively. (B and C) The remaining infected cells were washed and incubated in fresh medium for 40 hours before lysis. Luciferase activity was determined in equivalent amount of lysates. Each luciferase activity assay was done in duplicate and results are representative of 2 to 4 independent experiments. Mean CD4 expression for each cell line was determined before each assay and is indicated in parenthesis. R.L.U., relative luciferase units.

Next, we determined the efficiency of viral entry in these characterized A201 CD4 cell populations by means of two different entry assays. Infections were carried out with viruses produced from the chimeric proviral clones NL4, 3-luc-1558 and NL4, 3-luc-HXB that encode the luciferase reporter gene in place of *nef*. The *env *gene in these chimeric proviral constructs was subcloned respectively from the primary QH1558 and the laboratory-adapted HXB3 isolates, both of which are X4-tropic viruses [15]. Mean fluorescence values of CD4 expression at the surface of A201 CD4 cell lines were determined before each entry assay and are indicated in Figure 3.

In the first assay, viruses were produced by cotransfecting 293T cells with proviral DNA and an expression vector encoding a β-lactamase-Vpr (BlaM-Vpr) fusion protein. After infection with the BlaM-Vpr-containing virions, A201 cells were loaded with the CCF2-AM dye, a fluorescent substrate of BlaM, as previously described [16]. The cleavage of CCF2 results in a change of its fluorescence emission spectrum from green (520 nm) to blue (447 nm). The BlaM enzyme is active as soon as the virus enters cell, thus, allowing an early reading of viral entry by flow cytometry. Using the NL4, 3-luc-1558/BlaM-Vpr virus, we could observe, on average, an approximately two-fold increase in the percentage of infected cells for the A201 CD4-E91K, E92K and A201 CD4-Q344E cell populations as compared to A201 CD4-WT.

To further investigate the effect of CD4 dimers on viral infection, we assessed viral entry at a later stage of the replication cycle, by measuring luciferase activity in the remaining portion of infected cells, as previously described [15]. As the luciferase gene is encoded in place of the *nef *gene, it is expected that an increase in viral entry induces an increase in early viral transcription and consequently, in luciferase expression. Surprisingly, we did not observe a significant increase in luciferase activity with the A201 CD4-Q344E cell population as compared to A201 CD4-WT (Figure 3b), even though early measurement using BlaM-Vpr indicated a significant increase of viral entry with the A201CD4-Q344E cell population. In contrast, our results reveal that early viral transcription was significantly higher in A201 CD4-E91K, E92K cell line as compared to control cell lines (Figure 3c). In this last assay, we introduced a second A201 CD4-WT cell population, expressing higher levels of CD4, because CD4 cell surface expression in the A201 CD4-WT control cells had slightly decreased over time. Thus, to ensure that the difference observed in luciferase activities was not the result of a difference in CD4 expression, we compared the A201 CD4-E91K, E92K cell population with both A201 CD4-WT low and A201 CD4-WT high. Remarkably, despite lower expression of the D1 mutant, we could observe a four-fold increase in luciferase activity in A201 CD4-E91K, E92K compared to the A201 CD4-WT high cell population.

Next, we assessed whether disruption of CD4 dimerization had the same stimulatory effect on entry of a virus encoding envelope glycoproteins from a laboratory-adapted isolates, using the NL4, 3-luc-HXB chimeric provirus. The BlaM-Vpr assay revealed that NL4, 3-luc-HXB entry efficiency with the dimerization defective D1 mutant was increased given that it was comparable to that observed with a CD4-WT that is expressed at substantially higher levels (Figure 3d). A luciferase assay was also carried-out and indeed confirmed these observations (data not shown). All together, these results suggest that HIV-1 uses CD4 monomers more efficiently than CD4 dimers to mediate viral entry.

## Discussion

In this study, we provide evidence suggesting that disruption of CD4 dimerization has a positive effect on HIV-1 entry efficiency. The fact that we obtained similar results with two distinct CD4 dimerization mutants, targeting two different regions of the CD4 molecule suggest that the increase in entry efficiency is likely to result from the dimerization defect. Furthermore, given that we selected populations of CD4+ clones instead of single clones, the possibility that the observed increase in entry efficiency is the result of clonal effects is unlikely. Thus, we conclude that decreasing CD4 dimers levels might increase the efficiency of HIV-1 entry.

**Figure 4:**
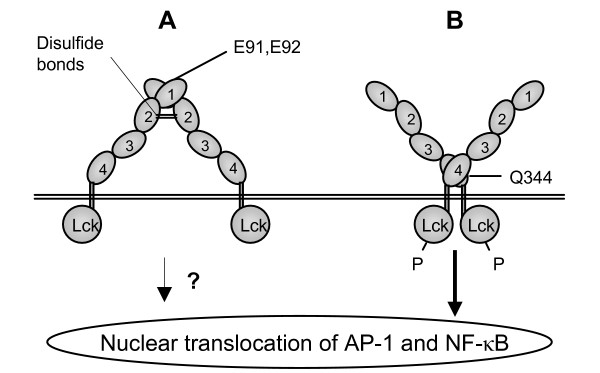
**Model of CD4 dimer conformations and their hypothetical difference in signal transduction via p56**^**lck**^. Usually, monomeric form of CD4 receptors is prevailing, but they can also dimerize, to some extent, through interactions of their D1 or D4 domains, resulting in two distinct dimer conformations. As crystallographic studies had only revealed the conformation B, it could be the predominant form of dimers. Critical residues in the dimer interfaces are depicted. This model suggest a different relative position of the cytoplasmic tails in the two CD4 dimer conformations that could affect their capacity to activate p56^lck ^and induce nuclear translocation of AP-1 and NF-κB.

The difference observed between the two dimerization mutants when entry was detected by measurement of early expression of the luciferase indicator gene could reflect a signalling defect specifically associated with the D4 mutant. Indeed, it was shown that CD4 dimerization induced by the binding of HIV-1 virions leads to the nuclear translocation of AP-1 and NF-κB [10]. As the LTR of HIV-1 contains binding sites for these transcription factors, their nuclear import is thought to increase early viral transcription [17,18]. Furthermore, the different phenotype obtained with both D1 and D4 mutants might indicate that two different forms of CD4 dimers may co-exist. Indeed, the structural characterization of the receptor, by crystallographic studies, has revealed a bent conformation that does not allow the simultaneous interaction between two D1 and two D4 domains [11]. Therefore, it is possible that D1 dimers do not lead to p56^lck ^activation as efficiently as D4 dimers because their cytoplasmic tail would be maintained at a significant distance. If this is the case, the D4 dimerization mutant would not induce AP-1 and NF-κB translocation in the nucleus as efficiently as the D1 mutant (Figure 4), resulting in reduced early viral expression. In accordance with this model, we have previously demonstrated that cells expressing the D4 mutant have an IL-2 secretion defect following SEB stimulation, suggesting a signalling deficiency in these cells [4]. However, other investigators have also shown a reduction in NF-κB nuclear translocation and in viral production for cells expressing the CD4-E91K, E92K dimerization mutant as compared with a CD4-WT expressing cell line [13]. These findings are not consistent with our results and further analysis are required to elucidate this discrepancy and to confirm our hypothesis.

Our results suggest that CD4 dimers have a negative effect on viral entry efficiency. Considering the low fraction of CD4 dimers expressed in the A201 CD4-WT cell population, it could appear surprising that a small pool of CD4 dimers could have such a significant effect on viral entry. However, given that several gp120-CD4-coreceptor complexes are required for the formation of a fusion pore as reported by several studies [19], a small fraction of a dimeric receptor may have a trans-dominant negative effect on viral entry efficiency. Furthermore, it has been demonstrated that HIV-1 binding to target cells induces the recruitment of CD4 and coreceptor to the so-called "virological synapse" [5,20,21]. Thus, the higher CD4 density at the site of viral entry could promote dimerization of the receptor and hence effectively affect the Env glycoproteins-mediated fusion process. Indeed, it has been shown that gp120 binding to CD4 increases dimerization of the receptor [22].

To date, the step at which CD4 dimers affect viral entry is not clear. The structural characterization of both mutants have indicated that E91K, E92K and Q344E mutations have only a local effect on the conformation of the receptor [4,13,23]. Moreover, a previous study has suggested that binding of gp120 was unaffected by the E91K, E92K mutations thus suggesting that gp120 interacts with CD4 dimers and monomers with similar efficiency [13]. Considering these previous findings, we propose that CD4 dimers mediate their viral entry inhibition by blocking a step between CD4 binding and membrane fusion. The most attractive hypothesis is that CD4 dimers could be less flexible than monomers. Indeed, it has been postulated that flexibility of the hinge regions of CD4 could be important for its HIV-1 receptor function [11,24]. Thus, CD4 dimers would bind to gp120, but would not bend as efficiently than CD4 monomers to allow the interaction between gp120 and its coreceptor. Further investigations are required to identify precisely the step affected by CD4 dimers. These studies should lead to a better understanding of processes underlying HIV entry, a step of the HIV-1 infection cycle that represents a validated target for antiretroviral therapy.

## Conclusion

Overall, our results suggest an association between disruption of CD4 receptor dimerization and increased HIV-1 entry efficiency. We propose that monomeric forms of CD4 are preferentially used by HIV-1 to gain entry into target cells. This implies that the dimer/monomer ratio at the cell surface of HIV-1 target cells may modulate the efficiency of HIV-1 entry.

## Methods

### Cells

Human SV40-transformed 293-T fibroblasts and HEK 293 cells were maintained in Dulbecco's modified Eagle's medium supplemented with 10% fetal calf serum (FCS) and 1% antibiotics (penicillin and streptomycin) (Multicell). A201 is a CD4 negative human T cell line derived from the CEM T cell line [14]. It was cultured in RPMI-1640 medium supplemented with 10% FCS and 1% antibiotics. A201 CD4 cell populations were produced by electroporation of A201 cells with a CD4 expression plasmid encoding CD4-WT or dimerization mutants of CD4, under the control of the LTR from the lymphotropic SL3 murine retrovirus [25], and encoding a hygromycin resistance gene. Populations of CD4-positive clones were selected by immunofluorescence cell sorting and cultured in RPMI-1640 medium supplemented with 10% FCS, 1% antibiotics and 0, 5 mg/ml hygromycin (Calbiochem).

### Immunofluorescence cell sorting and flow cytometry analysis

Cells were washed in PBS and incubated with the CD4 specific monoclonal antibody (mAb) OKT4 or the anti-CXCR4 mAb 12G5 in 5% FCS-PBS. After 30 minutes at 4°C, cells were washed twice in PBS and incubated for 30 minutes at 4°C with a fluorescein isothiocyanate (FITC)-conjugated goat anti-mouse Ab (BD Biosciences Pharmingen) in 5% FCS-PBS. Cells were then washed twice in PBS and CD4-positive clones were selected by immunofluorescence cell sorting using a MoFlow instrument (Cytomation, Fort Collins, CO) or analyzed by flow cytometry using a FACSCalibur cytometer (BD Biosciences) and plotted using the Cell Quest Pro software. As negative control for the CXCR4 staining, cells were stained with the FITC-conjugated secondary Ab only.

### Immunoprecipitation and western blot analysis

HEK 293 cells were transfected with SVCMV-CD4-WT, SVCMV-CD4-E91K, E92K and SVCMV-CD4-Q344E expression vectors by a calcium phosphate method or untransfected. Cells were harvested in PBS and lysed at 4°C during 30 minutes in a Nonidet-P40 (NP40)/Triton X-100 lysis buffer (1% NP-40/Triton X-100, 10 mM Hepes (pH 7, 5), 0, 14 M NaCl, 10 mM MgCl_2_, 10 mM EGTA, 10 mM NEM, 1 mM PMSF and a complete cocktail of protease inhibitor (Roche)) 48 hours post-transfection. Equivalent amounts of cell lysates were separated by SDS-PAGE, in a non-reducing Laemmli buffer (60 mM tris (pH 6, 8), 20 μg/ml bromophenol blue and 5% glycerol) as described previously [4], and transferred onto a nitrocellulose membrane (Bio Rad Laboratories). Immunoblot analysis was performed using a CD4-specific polyclonal Ab (Santa Cruz Biotechnology) as described previously [26].

A201 and the different A201 CD4 cell populations were lysed at 4°C during 30 minutes in a NP40/Triton X-100 lysis buffer. Cell lysates were normalized for total CD4 amounts and then immunoprecipitated with the anti-CD4 mAb OKT4 as described previously [26]. The immunocomplexes were resuspended in non-denaturing Laemmli buffer (60 mM tris (pH 6, 8), 20 μg/ml bromophenol blue, 5% glycerol and 2% SDS) and separated by SDS-PAGE. Immunoblot analysis was then carried out as described for the HEK 293 cells.

### Viral production

pNL4, 3-luc-1558 and pNL4, 3-luc-HXB are two reporter HIV-1 clones provided by B. Cullen (Duke University Medical Center, Durham, North Carolina) in which the *nef *gene has been replace by the luciferase gene and the *env *gene has been subcloned respectively from the primary isolate QH1558 and the laboratory-adapted isolate HXB3, both of which are X4 isolates [15]. HIV-1 virions containing the β-lactamase-Vpr (BlaM-Vpr) fusion protein were produced by transient transfection of 2 × 10^6 ^293T cells with 10 μg of proviral DNA and 5 μg of the pMM310 plasmid (Merck Research Laboratories, West Point, PA. USA), which encodes the BlaM-Vpr fusion protein, by the calcium-phosphate method. Viruses were harvested 40 hours post-transfection. The supernatant was clarified by centrifugation at 3000 rpm for 30 minutes and virions were concentrated by ultracentrifugation at 35000 rpm in a Beckman 70 Ti rotor for 1, 5 hour at 4°C. Virions were then resuspended in RPMI-1640 medium supplemented with 10% FCS and stored at -80°C. Virus level was quantified by performing a standard reverse transcriptase (RT) activity assay, as previously described [27].

### Virus-cell fusion assay

500 000 cells were incubated 3 hours with increasing viral inputs of BlaM-Vpr containing virions, in 500 μl RPMI-1640 medium. Cells were then extensively washed to remove free virions and incubed 1 hour, at room temperature, with CCF2-AM loading mix, as recommended by the manufacturer (Invitrogen). Next, exceeding dye was washed away and cells were incubated for 16 hours, at room temperature, prior to fixation with paraformaldehyde 4% (Canemco & Marivac). The percentages of infected cells were determined by flow cytometry, using a FACSVantage SE instrument (BD, Biosciences).

### Luciferase assay

500 000 cells were incubated 3 hours with increasing viral inputs in 500 μl RPMI-1640 medium. Cells were then extensively washed to remove free virions and incubated for 40 hours before lysis. Luciferase activity in equivalent amount of lysates was measured with an Auto Lumat LB 953 instrument (EG&G Bertho) by adding 100 μl of luciferase assay reagent (Promega).

## Competing interests

The author(s) declare that they have no competing interests.

## Authors' contributions

RB participated to all the experiments and contributed to writing of the manuscript. JM participated in the establishment of cell populations and provided crucial technical help for functional studies. IPB participated in the immunoblot analysis. EAC designed the study, participated to data analysis and contributed to writing of the manuscript. All authors have read and approved the manuscript.
